# Comparative transcriptomics revealed differential regulation of defense related genes in *Brassica juncea* leading to successful and unsuccessful infestation by aphid species

**DOI:** 10.1038/s41598-020-66217-0

**Published:** 2020-06-29

**Authors:** Lianthanzauva Duhlian, Murali Krishna Koramutla, S. Subramanian, Rohit Chamola, Ramcharan Bhattacharya

**Affiliations:** 1 ICAR-National Institute for Plant Biotechnology, Indian Agricultural Research Institute Campus, New Delhi, 110012 India; 2 Division of Entomology, Indian Agricultural Research Institute, New Delhi, 110012 India

**Keywords:** Molecular biology, Plant sciences

## Abstract

Productivity of Indian mustard (*B. juncea*), a major oil yielding crop in rapeseed-mustard group is heavily inflicted by mustard aphid, *L. erysimi*. Mustard aphid, a specialist aphid species on rapeseed-mustard crops, rapidly multiplies and colonizes the plants leading to successful infestation. In contrary, legume specific cowpea aphid, *A. craccivora* when released on *B. juncea* plants fails to build up population and thus remains unsuccessful in infestation. In the present study, differential host response of *B. juncea* to the two aphid species, one being successful insect-pest and the other being unsuccessful on it has been studied based on transcriptome analysis. Differential feeding efficiency of the two aphid species on mustard plants was evident from the amount of secreted honeydews. Leaf-transcriptomes of healthy and infested plants, treated with the two aphid species, were generated by RNA sequencing on Illumina platform and *de novo* assembly of the quality reads. A comparative assessment of the differentially expressed genes due to treatments revealed a large extent of overlaps as well as distinctness with respect to the set of genes and their direction of regulation. With respect to host-genes related to transcription factors, oxidative homeostasis, defense hormones and secondary metabolites, *L. erysimi* led to either suppression or limited activation of the transcript levels compared to *A*. *craccivora*. Further, a comprehensive view of the DEGs suggested more potential of successful insect-pests towards transcriptional reprogramming of the host. qRT-PCR based validation of randomly selected up- and down-regulated transcripts authenticated the transcriptome data.

## Introduction

Rapeseed–mustard group of crops constitute important sources of edible oil and leafy vegetables consumed worldwide. In this group, *Brassica juncea* or Indian mustard is grown as a major oilseed and leafy vegetable in Europe, Africa, North America, and parts of Asia including India. Like many of the *Brassica* spp. the productivity of *B. juncea* is severely constrained by damage due to mustard aphid, *Lipaphis erysimi*. Mustard aphid, a hemipteran sap-sucking insect is a specialist aphid species which has evolved as the most widespread and devastating insect-pest of *B. juncea*^1,2^. Equipped with specialised feeding mechanism and mode of reproduction it rapidly colonizes the plants and causes excessive diversion of phloem sap. A major constraint in protecting crop damage against aphid is the lack of genetic resistance which necessitates chemical based control through massive application of systemic insecticides.

In nature there are more than 4700 aphid species in 25 aphid subfamilies^3^. The host range of aphid species is widely diversified. Based on their host diversity, aphids have been grouped into mono-, oligo- and polyphagous in nature. Monophagous aphids tend to feed on one specific host, whereas oligophagous on few and polyphagous on many plant species during their life cycle^3^. For example, the green peach aphid, *Myzus persicae* has a wide range of hosts while its close relative pea aphid, *Acyrthosiphon pisum* has a limited number of hosts within the leguminous plants. The determinants of such differences in host range within the aphid species remain elusive^4,5^. Finding a specific host by a winged aphid involves a series of complex behavioural events between plant and aphids such as host finding, landing, probing etc. before the establishment of uninterrupted feeding. A small number of plant species can kill or repel aphids by toxic compounds secreted by the glandular trichomes^5^. Aphids are likely to use a phototactic visual response and phytochemical cues in determining the host^6^. For example, soybean aphid*, Aphis glycines* identifies soybean over the other nonhost plants through olfactory chemical signalling and it is evident as interruption by nonhost plants through odours decreases their ability to locate and colonize the host plants^7^.

During feeding from the sieve tube elements in phloem, aphids introduce effector molecules into the host cells for suppression of the host defense responses^4,8,9^. In the past decade, several effector molecules and their role in aphid virulence have been identified by either over-expression or suppression studies in the host plants. For example, over-expression of *M. persicae* effectors Mp10, Mp42, Mp56, Mp57, and Mp58 in Arabidopsis and tobacco plants reduced aphid virulence^10,11^. In contrary, similar over expression of a pea aphid C002 ortholog from *M. persicae* MpC0002 and other *M. persicae* effectors such as Mp1/PIntO1 and PIntO2 were found to be involved in increased virulence^9,10^. Further, it has been demonstrated that the effectors’ interaction with host proteins is species-specific. For example, the *M. persicae* Mp1 effector interacts with the host protein VPS52 to promote aphid virulence; whereas over-expression of potato StVPS52 in tobacco plants significantly reduced *M. persicae* fecundity^12^. Similarly, host plants over-expressing pea aphid effector C002 did not affect the performance of *M. persicae*^9^. Such impasse did not allow to hypothesize more universal role of any effector which could be targeted for devising resistance strategy against aphids.

Broad-spectrum, nonhost resistance against pathogens involves recognition and activation of the plant immune system. In contrary, the host defense system is either suppressed or evaded by effectors in case of compatible host-pathogen interactions^13–15^. Significant progress has been made on understanding how the plants respond to pathogens in interacting as a host or nonhost. Opposing to that, not much is known in understanding the plant-response as nonhost in case of plant-insect or plant-aphid interactions. Jaouannet *et al*.^16^ through microarray analysis demonstrated set of genes specifically affected during host or nonhost interactions with specific aphid species. However, because of limited evidence, lack of more studies and validation, our knowledge of nonhost resistance mechanisms in plant-aphid interactions remains limited. More recently, it was shown that BAK1 (Brassinosteroid insensitive 1-associated kinase 1), a key regulator of several leucine-rich repeat-containing PRRs (pattern recognition receptors) is involved in nonhost resistance to aphids. The pea aphid, *A. pisum* for which Arabidopsis is normally a nonhost survive better on *bak1-5* mutant plants suggesting that BAK1 contributes to nonhost resistance^17^. Similarly, bird-cherry oat aphid, *Rhopalosiphum padi* for which Arabidopsis is a nonhost survive longer on *vsp1* and *atrbohF-3* mutant plants, indicating that VSP1 and AtRbohF contributes to nonhost resistance against this aphid^16^. However, similar role of these genes in case of other species is not known.

In the last decade, other than *M. persicae*–Arabidopsis interaction, a few more studies based on microarrays or RNA-seq analyses have been carried out in different plant species for elucidating plant-responses’ to aphid infestation^18–22^. These studies revealed involvement of ROS homeostasis, cell signalling and production of secondary metabolites as a major part of the host defense response against aphids in addition to components of primary metabolism of the host plants. However, in case of *Brassica* sp. which comprise a major group of economically affected crops only limited information is available on host and nonhost-response to aphids^23^. For overcoming such bottleneck, in the present study, we have made a comparative assessment of the transcriptional responses of *B. juncea* under compatible and incompatible interaction with aphid species *L. erysimi* and *Aphis craccivora*, respectively. Out of that comparative analysis, the differentially regulated genes involved in pathways related to host resistance have been highlighted.

## Results

### Feeding performance of aphid species on *B. juncea* as host and nonhost

**Figure 1:**
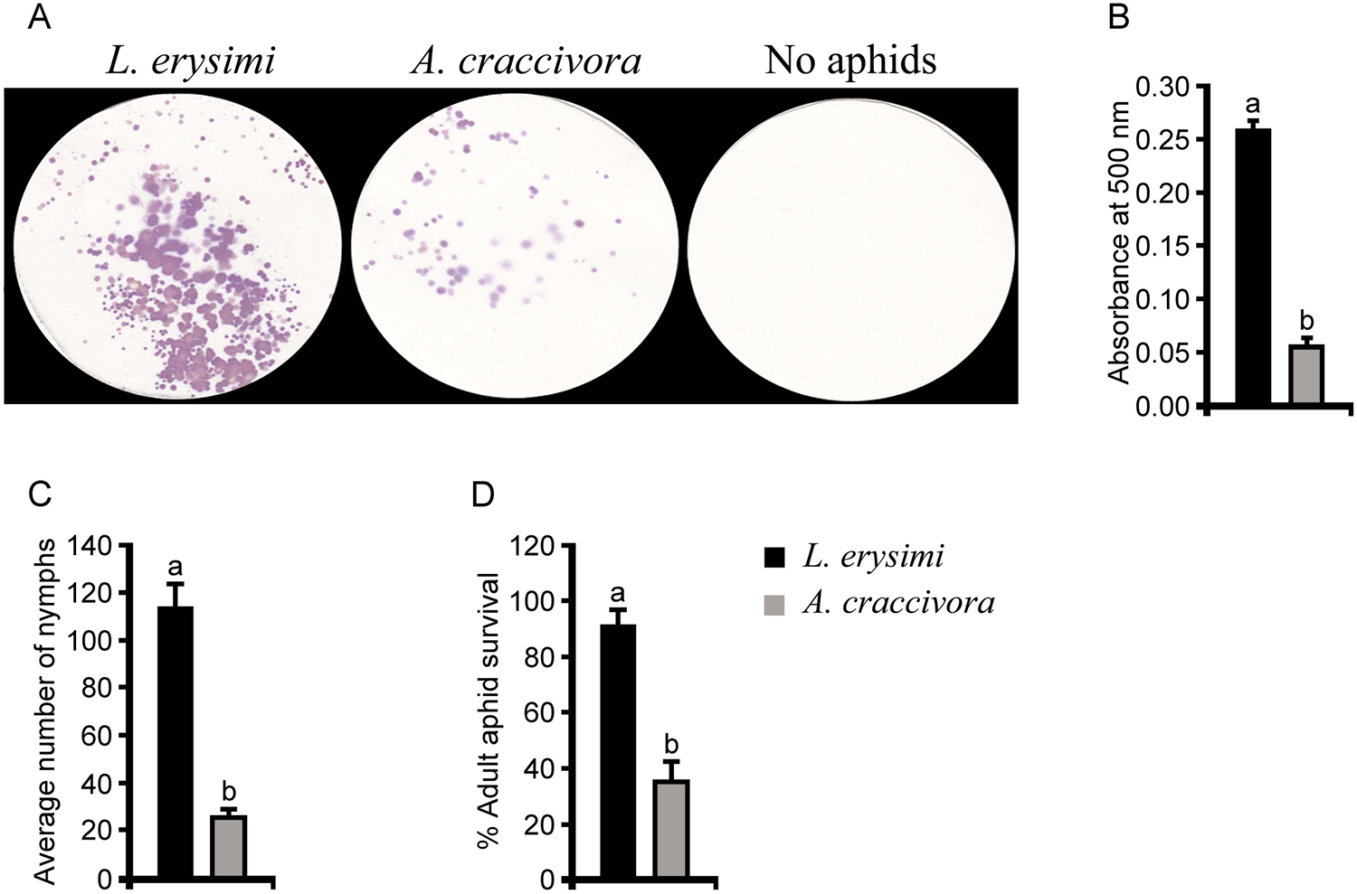
Feeding efficiency, survival and reproduction rate of *L. erysimi* and *A. craccivora* on *B. juncea*. (**A**) Ninhydrin staining of aphid honeydew after 24 h of feeding. (**B**) Quantification of honeydew by ninhydrin staining measured at λ_500_. (**C**) Fecundity of the two aphid species on mustard plants. (**D**) Survival rate of the two aphid species after 4 days of release. Bars represent means ± SE. Means with different letters are significantly different (Student’s *t* test, *p* < 0.05).

The feeding efficiency of an aphid species on a host is directly proportional to the amount of honeydew excretion^24^. Indian mustard, *B. juncea* is the natural host and a nonhost for the aphid species *L. erysimi* and *A. craccivora*, respectively. The feeding efficiency of *L. erysimi* and *A. craccivora* released on *B. juncea* plants was measured as a function of the amount of honeydew excreted by them in 24 h of feeding. The excreted honeydews collected on the Whatman paper discs were stained and quantified by Ninhydrin reagent^24^. The honeydews developed purple colour spots with different intensities after staining with ninhydrin. The number of purple spots produced in case of *L. erysimi*-feeding was much higher than the *A. craccivora-*feeding (Fig. 1A). Further, the spectrometric quantification of the spots revealed that, *L. erysimi* excreted 4.5 folds higher amount of honeydews compared to *A. craccivora* (Fig. 1B). Fecundity estimates of the aphids during the rearing period revealed that *L. erysimi* (host-aphid), produced nymphs at a rapid rate with an average of 114 nymphs per cage. On the other hand, the fecundity of *A. craccivora* (nonhost-aphid) was severely retarded with an average fecundity of 26 nymphs per cage (Fig. 1C). Similarly, the rate of survival of the adult *A. craccivora* was 2.5 folds lower than the rate of survival of *L. erysimi* adults (Fig. 1D). The results empirically suggested that the feeding and multiplication of *A. craccivora* was severely retarded on the plants of *B. juncea* which is not a natural host of it.

### Transcriptome sequencing, data records and *de novo* assembly

Whole transcriptome of *B. juncea* leaves independently infested with *L. erysimi* and *A. craccivora* were sequenced to identify the set of differentially regulated host-genes in case of successful and unsuccessful colonization. Sequencing was carried out on Illumina platform using 2 × 150 paired-end chemistry and the mean sizes of the inserts in the libraries were in the range of 450–675 bp. The raw reads obtained in uninfested (control), *L. erysimi-*infested (LE) and *A. craccivora*-infested (AC) samples were 5.7, 5.4 and 5.2 Gb, respectively (Table 1). All sequence reads were deposited in the National Center for Biotechnology Information (NCBI) Sequence Read Archive (accession SAMN12924495-SAMN12924497) under Bioproject PRJNA576081(https://www.ncbi.nlm.nih.gov/sra/ PRJNA576081). After removing the adaptor sequences, low quality reads and over-represented sequences, high-quality clean reads were obtained from the above three libraries. These high-quality reads were *de novo* assembled using CLC Genomics workbench 6.0 on optimized parameters which led to the identification of 48775, 49646 and 42182 transcripts in case of control, LE and AC samples. The minimum and maximum contig size was 500 bp and 16812 bp, respectively with an N50 value greater than 1000. The size distribution of transcript lengths has been shown in Supplementary Fig. S1. Further, the assembled transcript contigs were analysed for identifying coding regions which revealed a total of 47806, 48638 and 41389 CDS with varying length in control, LE and AC infested samples, respectively.

**Table 1:** Summary of sequencing and assembly data of control, LE and AC infested *B. juncea*.

	Control	LE	AC
Data output (Gb)	5.7	5.4	5.2
High quality reads	20606561	19387846	19321340
Number of transcripts	48775	49646	42182
Maximum transcript size (bp)	16812	8529	9223
N50	1053	1207	1182
Number of CDS	47806	48638	41389

### Functional annotation of aphid inducible transcriptome in *B. juncea*

**Figure 2:**
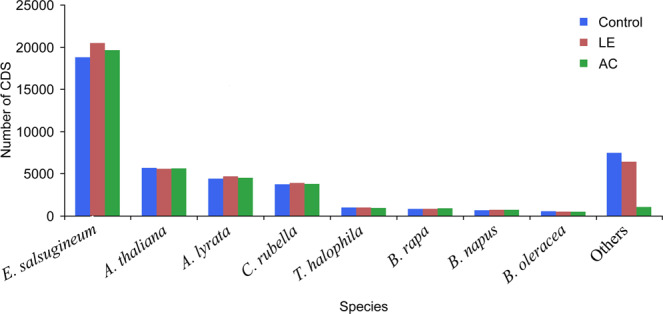
Species distribution of the top blastx matches following nr annotation of unigenes in leaf transcriptome of *Brassica juncea* treated with successful and unsuccessful aphid species.

The assembled transcripts were aligned with the non-redundant NCBI plant protein database using blastx with a cut-off E-value of 10^−6^. The number of CDS showing significant matches were 43485, 44507, 38068 and unmatched CDS were 5290, 5139, 4114 in control, LE and AC infested samples, respectively. The transcriptome data sets were also annotated against TAIR10 protein database. In TAIR blastx 39813, 41184, 37630 coding sequences in case of control, LE and AC infested samples, respectively found significant matches. Species distribution of the best match sequences suggested that majority of the hits were from the Brassicaceae members comprising *Eutrema salsugineum, Arabidopsis thaliana, A. lyrata,Capsella rubella*, *Thellungiela halophila*, *Brassica rapa*, *B. napus*, *B. oleracea* and *B. juncea* (Fig. 2). The transcriptome data sets also shared similarity with species outside the Brassicaceae family viz., *Phaseolus vulgaris* and *Glycine max*. Further, the annotated CDS were mapped on to the GO database for identifying nodes comprising of GO functional groups. CDS associated with similar functions are assigned to the same GO functional group. Based on the sequence homology, 30681 (Control), 31383 (LE) and 26088 (AC) CDS were grouped under Biological Process, 28877 (Control), 29468 (LE) and 24339 (AC) CDS were assigned under Molecular Function and 30929 (Control), 32028 (LE) and 26371 (AC) CDS were categorised under Cellular Component (see Supplementary files 1–3). All the CDS were also compared against the KEGG database (www.kegg.jp/kegg/kegg1.html) using blastx with threshold bit-score value of 60 (default)^25^. The mapped CDS represented metabolic pathways of major biomolecules such as carbohydrates, lipids, nucleotides, amino acids, glycans, cofactors, vitamins, terpenoids and polyketides etc. Details of the functional pathways and their sub-categories are provided in Table 2.

**Table 2:** KEGG pathway distribution of *B. juncea* leaf transcriptome.

Pathway	Control	LE	AC
**Metabolism**			
Carbon and fatty acid metabolism	519	570	512
Carbohydrate metabolism	591	631	572
Energy metabolism	556	561	503
Lipid metabolism	358	388	324
Nucleotide metabolism	226	247	220
Amino acid metabolism	483	517	476
Metabolism of other amino acids	195	218	182
Glycan biosynthesis and metabolism	112	131	112
Metabolism of cofactors and vitamins	287	317	299
Metabolism of terpenoids and polyketides	184	187	158
Biosynthesis of other secondary metabolites	156	163	159
Xenobiotics biodegradation and metabolism	116	123	83
**Genetic Information Processing**			
Transcription	334	345	312
Translation	879	970	806
Folding, sorting and degradation	676	700	621
Replication and repair	176	196	167
**Environmental Information Processing**			
Membrane transport	36	30	29
Signal transduction	551	616	550
Signalling molecules and interaction	1	1	4
**Cellular Processes**			
Transport and catabolism	390	392	340
Cell motility	58	65	60
Cell growth and death	212	230	202
Cell communication	64	69	64
**Organismal Systems**			
Environmental adaptation	210	214	207

### Differential gene expression analysis

**Figure 3:**
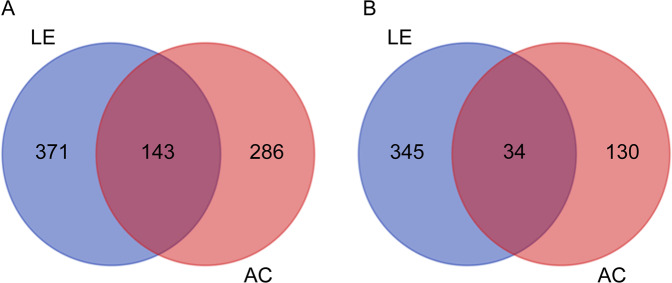
Venn diagram showing the common and unique differentially expressed genes (DEGs) in response to infestation by *L. erysimi* (LE) and *A. craccivora* (AC). (**A**) Up-regulated DEGs in response to LE and AC infestations. (**B**) Down-regulated DEGs in response to LE and AC infestations.

A common data set was generated for identifying differentially expressed transcripts in *B. juncea* infested with host (LE) and nonhost (AC) aphids. FPKM values were calculated for each condition and used to normalize the transcript expression. The FPKM values of aphid infested samples were compared with uninfested control samples for identifying the differentially expressed transcripts. Based on log2 ratio ≥2 and *P* < 0.05 threshold values, total 1307 genes were identified as differentially expressed in LE and AC infested samples when independently compared to the uninfested control (see Supplementary file 4). Out of 1307 genes, 514 and 429 genes were up-regulated whereas 379 and 164 genes were down-regulated in LE and AC samples, respectively. The results also showed overlapping of 143 up- and 34 down-regulated genes in LE and AC samples (Fig. 3). Analysis of gene ontology terms of the differentially expressed transcripts in LE and AC infested samples over the control was represented in Supplementary file 5 and Fig. S2. In case of up-regulated transcripts in both the treatments, significantly enriched GO terms in molecular function and biological processes were almost similar, representing response to abiotic stimulus (GO:0009628), cellular process (GO:0009987), response to stimulus (GO:0050896), developmental process (GO:0032502) and catalytic activity (GO:0003824). However, in AC-infested samples enriched GO terms represented defense response to bacterium (GO:0042742), hormone transport (GO:0009914) and carbohydrate metabolic process (GO:0005975). Further, in biological processes enriched GO terms of down-regulated transcripts in case of LE-infested sample distinctly represented defense related mechanisms such as secondary metabolic process (GO:0019748), response to oxidative stress (GO:0006979), phenylpropanoid biosynthetic process (GO:0009699), sulfur metabolic process (GO:0006790), defense response to bacterium (GO:0042742), defense response (GO:0006952) and glucosinolates biosynthetic process (GO:0019761), characteristically present in members of Brassicaceae.

**Figure 4:**
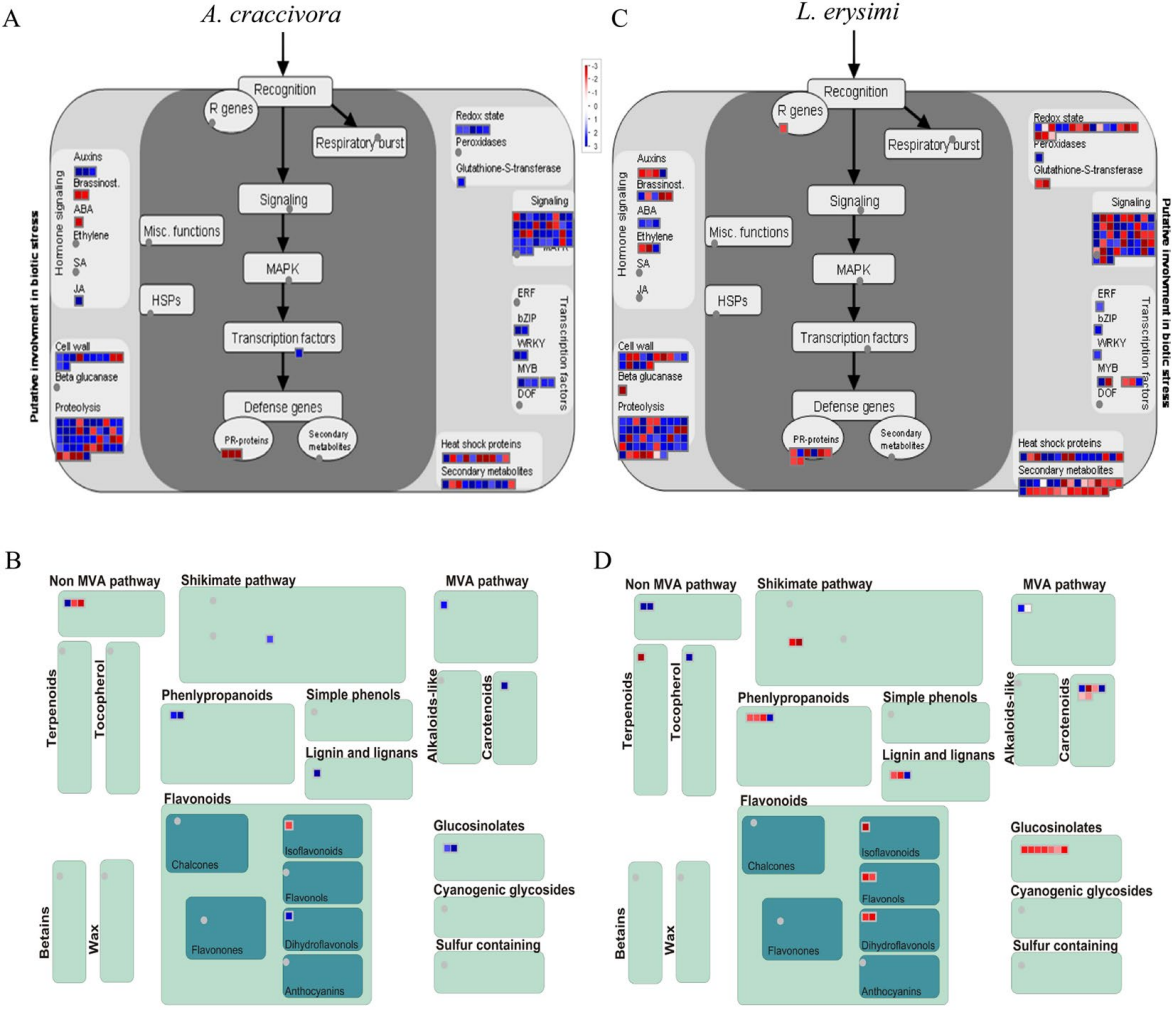
Differentially expressed genes in response to AC and LE in *B. juncea* assigned to biotic stress (**A,C**) and secondary metabolism (**B,D**) categories based on MapMan software^63^. The fold change in expression relative to uninfested control sample is indicated by blue (≥2 fold) and red (≥−2 fold) colors in the scale.

Further, to understand the mechanistic differences in AC and LE induced host responses, differentially expressed genes were analyzed by the MapMan software built in biotic stress and secondary metabolism overview under pathways. AC and LE infested transcriptome showed significant differences in biotic stress and secondary metabolism responses (Fig. 4). Under biotic stress, the transcripts belonging to hormone signalling, cell wall modification, proteolysis, redox state including glutathione S-transferase (GST), signalling, secondary metabolites, transcription factors, and heat shock protein categories were found to be up-regulated in response to AC (Fig. 4A,B). However, most of these pathways were suppressed within 24 h of successful host colonization by LE (Fig. 4C). Similarly, the transcripts belonging to biosynthetic pathways of secondary metabolites such as phenylpropanoid, carotenoids, flavonoids, lignin and glucosinolates were induced in response to AC (Fig. 4B), whereas attenuated by LE-infestation (Fig. 4D). The analysis empirically advocated suppression of defense related pathways in *B*. *juncea* in case of successful colonization by LE.

### Differentially expressed transcription factors in response to *L. erysimi* and *A. craccivora*

**Figure 5:**
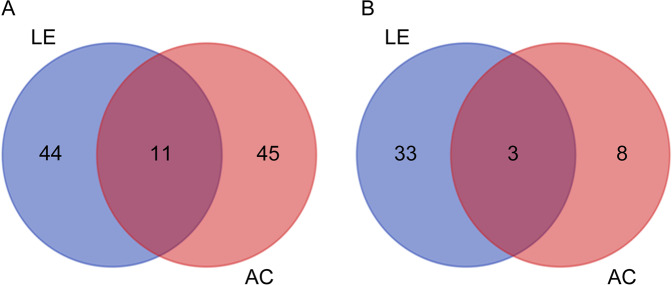
Venn diagram showing the common and unique Transcription Factors (TFs) differentially expressed in response to infestation by *L. erysimi* (LE) and *A. craccivora* (AC). (**A**) Transcription factors up-regulated in response to LE and AC infestations. (**B**) Transcription factors down-regulated in response to LE and AC infestations.

Transcription factors (TF) are the key regulatory proteins involved in modulating gene expressions. Out of the 1307 differentially expressed genes in LE and AC transcriptomes compared to uninfested controls, 158 genes were identified as encoding transcription factors (TFs). Out of 158 TFs, 91 were from LE and 67 from AC samples (see Supplementary file 6). Further analysis showed that most of the TFs were distinct to LE or AC library whereas, at the most 10% were overlapping (Fig. 5A). Further, a comparative assessment of down-regulated TFs showed that higher number of distinct TFs are suppressed or down-regulated in LE compared to AC samples and only 7% TFs were commonly down-regulated in both the samples (Fig. 5B). In structural grouping, the differentially expressed TFs were spanned over 32 families. Twenty-one family were common in both LE and AC, whereas 7 families (AP2, LBD, e2f-dp, ERF, G2-like, HB-other, GATA) were specific to LE and 4 families (Nin-like, CAMTA, M-type, TCP) were specific to AC. The major families of the differentially expressed TFs in LE sample were bHLH (11), GeBP (9), NAC (8), B3 (5) and CO-like (5). These 5 families of TFs represented 41.75% of total TFs differentially expressed in response to *L. erysimi*. In response to infestation by *A. craccivora*, bHLH (10), GeBP (6), and S1Fa-like (5) were the major TF-families.

### Analysis of oxidative stress related transcripts in *B. juncea* as host and nonhost to aphid infestations

Transient activation of redox related genes constitutes the secondary signalling in plant defense response^26^. The genes involved in transient generation as well as scavenging of free radicals determine the ROS homeostasis. The ROS homeostasis genes showing differential expression between LE vs uninfested and AC vs uninfested were further compared for identifying the set of genes in common as well as distinct and the direction of their regulation (Table 3). The analysis showed that quantitatively a greater number of distinct transcripts involved in scavenging process are up-regulated in *B. juncea* in response to *L. erysimi* compared to response against *A. craccivora*. In plants, small intracellular thiol molecules, glutathione is considered as a strong non-enzymatic scavenger of reactive oxygen species (ROS). It serves as a substrate to the glutathione peroxidase and glutathione *S*-transferase in scavenging process. We observed that several transcripts involved in glutathione metabolism such as glutathione S-transferase, ascorbate peroxidase and L-ascorbate oxidase were induced in case of AC sample. In case of LE-infestation though peroxidases and glutaredoxin family proteins were activated, many of the genes involved in redox homeostasis including *CAT2*, *MSD1* etc. were down-regulated. The results indicated limited activation of ROS scavenging enzymes leading to elevated levels of free radical production in *B. juncea* as nonhost response to *A. craccivora* compared to its susceptible response to *L. erysimi*.

**Table 3:** Differentially expressed transcripts related to oxidative stress in response to successful and unsuccessful *B. juncea*- aphid interactions.

Common hit ID	TAIR ID	log2 (LE/Control)	TAIR Description
BAF00793	at5g37830	4.35	Oxoprolinase 1 (OXP1)
XP_002875944	at3g49110	2.86	Peroxidase 33 (PER33)
AAD17935	at4g35090	−2.91	Catalase 2 (CAT2)
XP_006291823	at3g26060	−2.33	Antioxidant/peroxiredoxin
AAB60902	at3g10920	−2.48	Manganese superoxide dismutase 1 (MSD1)
XP_002870217	at4g15680	2.43	Glutaredoxin family protein
ESQ53851	at4g33040	2.11	Glutaredoxin family protein
XP_002887881	at1g64500	−2.10	Glutaredoxin family protein
XP_006294288	at2g41330	−3.11	Glutaredoxin family protein
XP_006298621	at3g15360	−2.61	Thioredoxin M-type 4 (TRX-M4)
AAP58393	at2g02930	−2.84	Glutathione S- transferase F3 (GSTF3)
BAJ33811	at3g03190	−2.26	Glutathione S- transferase F11 (GSTF11)
XP_002893406	at1g26340	2.34	Cytochrome b5 isoform A (Cb5-A)
ESQ39310	at2g46650	−2.63	Cytochrome b5 isoform C (Cb5-C)
ESQ49825	at3g02870	−2.86	L-galactose-1-phosphate phosphatase
NP_568360	at5g18120	2.49	APR-like 7 (APRL7)
ABB17025	at1g77510	2.31	Protein disulfide isomerase-like 1–2
AET14214	at4g26850	−2.00	Vitamin C defective 2 (VTC2)
**Common hit ID**	**TAIR ID**	**Log2 (AC/Control)**	**TAIR Description**
AAN60795	at1g07890	2.50	Ascorbate peroxidase 1 (APX1)
ESQ42133	at5g21105	3.10	L-ascorbate oxidase
NP_172147	at1g06620	2.01	2-oxoglutarate-dependent dioxygenase
NP_177955	at1g78320	2.44	Glutathione S- transferase tau 23 (GSTU23)
ESQ47277	at5g37830	2.25	Oxoprolinase 1 (OXP1)
NP_568360	at5g18120	2.08	APR-like 7 (APRL7)
ABB17025	at1g77510	2.72	Protein disulfide isomerase-like 1–2

### Analysis of phytohormone-related transcripts

Phytohormones are known to play important role in plant defense against various biotic and abiotic stresses. Therefore, we investigated the differential transcript activation of the genes related to phytohormone biosynthetic pathways in *B. juncea* transcriptome in response to *L. erysimi* and *A. craccivora*. The analysis indicated differential expression of genes related to primarily six phytohormones viz., auxin (9 genes), abscisic acid (ABA; 4 genes), ethylene, (ET; 4 genes), jasmonic acid, (JA; 3 genes), salicylic acid (SA; 1 gene) and brassinosteroid (BR; 7 genes) (Table 4). Among the transcripts involved in auxin homeostasis*UGT75B*, *PIN4* and *UGT74B1* were down-regulated in LE-infested samples, whereas *TIR3* and *ARF18* showed up-regulation in both LE and AC infested samples. Interestingly, *LAX3, PIN7* and *ARF2* were specifically up-regulated in AC-infested sample. Three transcripts *ABF4*, *KOB1*, *NCED4* which are involved in ABA signalling were up-regulated in the LE transcriptome, while ABA-responsive element binding protein3 (*AREB3*) transcripts were down-regulated in AC transcriptome. ET signalling pathway related genes such as *multiprotein bridging factor 1C* (*MBF1C*) and *cooperatively regulated by ethylene and jasmonate1*(*CEJ1*) were down-regulated and *DEAD box RNA helicase 20* (*RH20*) was up-regulated in case of LE. In contrary, EIN3-binding F-box protein2 (*EBF2*) was up-regulated in case of AC set.

**Table 4:** Differentially expressed genes related to phytohormone in response to successful and unsuccessful *B. juncea*-aphid interactions.

	Common hit ID	TAIR ID	log2 (LE/Control)	TAIR Description
Auxin	AAL09350	at1g24100	−2.44	UDP-glucosyl transferase 74B1 (UGT74B1)
	BAD93921	at2g01420	−2.58	PIN-FORMED4 (PIN4)
	BAJ33712	at1g05560	−2.15	UDP-Glucosyltransferase 75B1 (UGT75B1)
	ESQ49882	at3g02260	3.06	Transport inhibitor response3 (TIR3)
	AFD01316	at3g61830	2.52	Auxin response factor18 (ARF18)
	ESQ33837	at1g30330	2.02	Auxin response factor6 (ARF6)
ABA	ESQ47969	at3g19290	2.06	ABRE binding factor4 (ABF4)
	NP_187467	at3g08550	2.68	KOBITO (KOB1)
	BAJ34120	at4g19170	2.27	Nine-cis-epoxycarotenoid dioxygenase4 (NCED4)
ET	XP_002876019	at3g50260	−2.26	Cooperatively regulated by ethylene and jasmonate1(CEJ1)
	ESQ29967	at1g55150	2.64	DEAD box RNA helicase, putative (RH20)
	NP_189093	at3g24500	−3.60	Multiprotein bridging factor 1C (MBF1C)
JA	AAN51933	at1g19670	−2.92	Coronatine-induced protein1 (CORI1)
	ADJ58019	at2g29980	3.09	Fatty acid desaturase3 (FAD3)
SA	ESQ39613	at5g45110	−3.21	Regulatory protein NPR3 (NPR3)
BR	ESQ36868	at2g07050	2.63	Cycloartenol synthase1 (CAS1)
	ESQ54135	at4g30610	−2.72	BRI1 suppressor1 (BRS1)
	BAJ34430	at4g33430	−3.90	BRI1-ASSOCIATED RECEPTOR KINASE (BAK1)
	AFU83230	at4g39400	2.13	BRASSINOSTEROID INSENSITIVE1 (BRI1)
	**Common hit ID**	**TAIR ID**	**Log2 (AC/Control)**	TAIR **Description**
Auxin	ESQ49882	at3g02260	2.34	TIR3
	XP_002862319	at1g77690	3.30	LIKE AUX1 3 (LAX3)
	ESQ34480	at1g23080	4.32	PIN-FORMED7 (PIN7)
	AFD01316	at3g61830	2.42	Auxin response factor18 (ARF18)
	AFD01294	at5g62000	2.22	Auxin response factor2 (ARF2)
ABA	ESQ44396	at3g56850	−2.71	ABA-responsive element binding protein3 (AREB3)
ET	BAJ34438	at5g25350	2.69	EIN3-binding F-box protein2 (EBF2)
JA	BAJ34294	at1g76690	2.98	12-oxophytodienoate reductase2 (OPR2)
BR	ESQ45459	at3g50750	−2.41	Brassinosteroid signalling positive regulator-related
	ESQ31959	at5g24150	−2.68	Squalene monooxygenase
	O65726	at5g24150	−2.95	Squalene monooxygenase

JA and SA-related genes in *B. juncea* also showed differential regulation when the response against *L. erysimi* and *A. craccivora* was compared (Table 4). The expression of *NPR3*, a paralog of NPR1 involved in SA signalling was down- regulated in LE infestation. In case of JA pathway, the transcripts encoding fatty acid desaturase3 (FAD3) was up- and coronatine-induced protein1 (COI1) was down-regulated in LE infested sample. COI1 is involved in signal transduction process of JA signalling. Thus, the result indicated likely suppression of JA signalling pathways in *B. juncea* in case of *L*. *erysimi* infestation. A similar suppression was not detected in case of AC sample which rather showed an increase in transcript level of *12-oxophytodienoate reductase2* (*OPR2*) involved in JA biosynthetic pathway. Transcripts encoding BR-related pathway such as Brassinosteroid signalling positive regulator (BZR1) family protein and squalene monooxygenase were down-regulated in AC. Whereas the transcript *BRI1 suppressor1* (*BRS1*), a serine carboxypeptidase and *BRI1-associated receptor kinase1* (*BAK1*) were down-regulated and *BRASSINOSTEROID INSENSITIVE1* (*BRI1*), a brassinosteroid receptor was up-regulated in LE-infested samples (Table 4).

### Transcript response related to secondary metabolite biosynthesis

Plants synthesize secondary metabolites such as phenols, terpenes, flavonoids, sulphur and nitrogen containing compounds to combat against microbial infections and insect herbivory^27^. In the transcriptome data, several transcripts showing differential level were mapped to the genes involved in the synthesis of various secondary metabolites belonging to isoprenoid, flavonoid, carotenoid, lignin and glucosinolates. A total of 36 transcripts involved in secondary metabolite production were differentially expressed in LE and AC infested samples compared to their uninfested controls (Table 5). The transcript responses against the two aphid species with respect to genes involved in secondary metabolism consist mostly of common set of genes and with similar pattern of regulation (Table 5). Nevertheless, the transcripts which were distinct in LE and AC were also identified. Interestingly, the direction of regulation of the differentially expressed genes in case of *A. craccivora* infested sample (AC), was essentially activation type; whereas the majority of differentially expressed genes showed down-regulation in case of LE infested plants. For example, all the differentially expressed flavonoid biosynthetic genes were down-regulated in LE, but not in AC-infested sample (Table 5).

**Table 5:** Differentially expressed genes related to secondary metabolite biosynthesis in *B. juncea* in response to infestation by *L. erysimi* and *A. craccivora*, respectively.

	Common hit ID	TAIR ID	log2 (LE/Control)	TAIR Description
Terpenoids biosynthesis	AAK59424	at4g15560	−3.68	Cloroplastos alterados1 (CLA1)
	ESQ55938	at4g15560	4.73	Cloroplastos alterados1 (CLA1)
	XP_006283682	at4g34350	3.02	4-hydroxy-3-methylbut-2-en-1-yl diphosphate reductase (ISPH)
	BAJ33798	at4g34350	−2.46	4-hydroxy-3-methylbut-2-en-1-yl diphosphate reductase (ISPH)
	ESQ29159	at3g54250	2.37	Mevalonate diphosphate decarboxylase, putative
	ESQ30852	at5g23960	−3.26	Terpene synthase21 (TPS21)
Flavonoid biosynthesis	CAP09039	at5g08640	−2.08	Flavonol synthase (FLS)
	ESQ37977	at5g63590	−2.49	Flavonol synthase3 (FLS3)
	ABB91635	at3g51240	−2.58	Flavanone 3-hydroxylase (F3H)
	BAJ34425	at1g75280	−2.82	Isoflavone reductase, putative
	NP_200210	at5g53990	−2.24	Glycosyltransferase family protein
Carotenoid biosynthesis	AEX31291	at5g17230	3.11	Phytoene synthase (PSY)
	AGZ62518	at1g06820	2.74	Carotenoid isomerase (CRTISO)
	ACS45170	at4g25700	−3.51	Beta-hydroxylase1 (BETA-OHASE1)
Lignin biosynthesis	AAG14961	at4g36220	−2.32	Ferulic acid 5-hydroxylase1 (FAH1)
	ESQ53188	at4g39330	2.82	Cinnamyl alcohol dehydrogenase9 (CAD9)
	ADC40029	at1g15950	−2.03	Cinnamoyl coA reductase1 (CCR1)
	ESQ33074	at2g02400	−2	Cinnamoyl-CoA reductase family
Glucosinolate biosynthesis	ACR10244	at3g19710	−2.32	Branched-chain aminotransferase4 (BCAT4)
	AAL09350	at1g24100	−2.44	UDP-glucosyl transferase 74B1 (UGT74B1)
	ACR10252	at1g16410	−2.23	Cytochrome P450 79F1 (CYP79F1)
	AGD95055	at4g13770	−2.14	Cytochrome P450 83A1 (CYP83A1)
	ACR10273	at1g18590	−2.23	Sulfotransferase17 (SOT17)
Glucosinolate breakdown	ESQ51834	at2g33070	4.86	Nitrile specifier protein2 (NSP2)
Tyrosine breakdown	ESQ50444	at5g36160	3.01	Aminotransferase-related
	Common hit ID	TAIR ID	log2 (AC/Control)	TAIR Description
Terpenoids biosynthesis	ESQ50058	at2g26930	−2.1	4-(CYTIDINE 5′-PHOSPHO)-2-C-METHYL-D-ERITHRITOL KINASE (CDPMEK)
	BAF81514	at1g63970	−2.7	2-C-methyl-D-erythritol 2,4-cyclodiphosphate synthase (ISPF)
	ESQ32252	at5g27450	2.38	MEVALONATE KINASE (MK)
	ESQ55938	at4g15560	4.31	Cloroplastos alterados1 (CLA1)
Flavonoid biosynthesis	ABY89688	at5g07990	2.75	Transparent testa7 (TT7)
	BAJ34425	at1g75280	−2.1	Isoflavone reductase, putative
Carotenoid biosynthesis	AGZ62518	at1g06820	3.24	Carotenoid isomerase (CRTISO)
Lignin biosynthesis	ESQ53188	at4g39330	3.77	Cinnamyl alcohol dehydrogenase9 (CAD9)
	NP_177876	at1g77520	2.36	O-methyltransferase family 2 protein
Glucosinolate breakdown	CAA79989	at5g26000	2.03	Thioglucoside glucohydrolase1 (TGG1)
	ESQ51834	at2g33070	4.99	Nitrile specifier protein2 (NSP2)

Several transcripts showing differential expression in both LE and AC data set were mapped to glucosinolate (GSL) metabolism. Our results showed that all the differentially expressed transcripts annotated for GSL biosynthesis such as branched-chain aminotransferase4 (BCAT4), cytochrome P450 79F1 (CYP79F1), cytochrome P450 83A1 (CYP83A1), UDP-glucosyl transferase 74B1 (UGT 74B1), and Sulfotransferase17 (SOT17) were down-regulated in case of LE infestation. This clearly indicated attenuating effect of *L. erysimi* on GSL biosynthesis leading to susceptible plant-aphid interaction. In contrary, enzymes involved in GSL breakdown such as nitrile-specifier protein 2 was up-regulated in case of both LE and AC, while thioglucoside glucohydrolase1 (TGG1) was up-regulated only in AC-infested sample (Table 5). Several hydrolases are involved in breakdown of glucosinolates into more toxic compounds related to defense. Taken together, the results show that the *L. erysimi* (host) infestation suppressed many of the secondary metabolite biosynthetic genes, more profoundly GSL biosynthetic genes; whereas the number of related transcripts showing activation was more in case of *A. craccivora* infestation.

### Validation of transcripts of RNA-seq data by qRT-PCR

**Figure 6:**
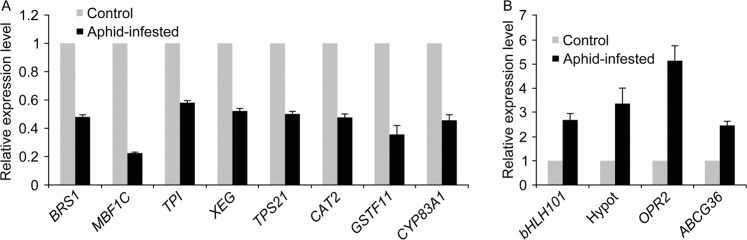
qRT-PCR based validation of differentially expressed transcripts. (**A**) Transcripts down-regulated in LE infested sample compared to uninfested control. (**B**) Transcripts up-regulated in LE (*bHLH101*, Hypothetical protein) and AC (*OPR2*, *ABCG36*) infested samples when compared to uninfested control.

For authenticating the transcriptomic data, randomly twelve differentially expressed genes including eight down-regulated transcripts (*BRS1, MBF1C, TPS21, XET, CAT2, CYP83A1, GSTF11*, trypsin and protease inhibitor family protein) and four up-regulated transcripts (*bHLH101, OPR2, ABCG36*, hypothetical protein) in LE and AC samples were selected. All the eight selected down-regulated genes in LE-infested samples showed similar expression pattern of down-regulation in qRT-PCR based validation when compared with their expression level in uninfested control (Fig. 6A). Similarly, the selected up-regulated transcripts in LE and AC showed an induced expression of about 2.4 to 5 folds higher in the host and nonhost aphid infested samples as compared with their respective un-infested controls (Fig. 6B). Thus, the qRT-PCR results confirmed reliability and quality of the transcriptome data and the estimation of genes expressions based on FPKM values.

## Discussion

Mustard aphid, *L. erysimi* is a specialized aphid species which heavily infests most of the rapeseed-mustard members including Indian mustard, *B. juncea*. Quantitative resistance to mustard aphid is unavailable among the cultivated germplasms of rapeseed-mustard^28^. Mustard aphid has evolved with mechanisms for attenuating defense machinery of the host plants and rapid colonization^23^. In contrary, the legume specialist cowpea aphid *Aphis craccivora* fails to establish a rapid infestation on *B. juncea* which is not a natural host. In the present study, differential host response of *B. juncea* to two different aphid species, one being successful insect-pest and the other being unsuccessful on it have been studied. In case of unsuccessful interaction, elicitation of endogenous defense and downstream metabolic changes limit the insect herbivores^29^. Feeding fitness of the two aphid species on *B. juncea* plants was assayed by quantification of honeydews based on ninhydrin staining (Fig. 1). The results showed significantly low rates of phloem diversion by *A. craccivora* compared to *L. erysimi* when released on *B. juncea* plants. Retarded feeding also led to low survival rates and highly inhibited fecundity of *A. craccivora* compared to *L. erysimi*. Earlier studies showed that on nonhost plants aphids do probe, survive and reproduce but only to a limited extent. For example, about 50% of *A. pisum* and 60% of *R. padi* adult aphids survived for 3 to 4 days in survival experiments on nonhost Arabidopsis plants^16,17^. Molecular responses of *B. juncea* against two of these aphid species *L. erysimi* (sample LE) and *A. craccivora* (sample AC) were assessed in terms of changes in transcriptome of *B. juncea* leaves at 24 h post infestation with reference to key defense related pathways.

Statistical analysis of the transcriptome data identified a large number of differentially expressed genes related to plant-aphid interactions^30^. Overall, in *L. erysimi* infested sample (LE) the number of up- and down-regulated transcripts were more compared to the *A. craccivora* infested sample (AC) indicating that more quantitative molecular response involved in *B. juncea*-*L.erysimi* interaction. It also suggested more potential of successful insect pests towards transcriptional reprogramming of host^31,32^. The two aphid species altered the gene-expression of several host transcription factors (Supplementary file 6), which are the important regulators of gene-expression during various biotic and abiotic stresses^33,34^.Both the aphid species up-regulated the bHLH TF family transcripts such as MYCs which play an important role in JA-mediated plant defense against herbivores and pathogens^34^. NAC transcription factors play diverse roles in response to biotic and abiotic stresses and in growth and development^35^. For example, transcripts of Arabidopsis *ATAF1* were increased in response to drought, high salinity, ABA, JA, wounding and *Botrytis cinerea* infection^36^. Our results also showed the involvement of NAC TF family in plant-aphid interaction which further influence the expression of several transcripts. Further, accumulation of starch plays a defensive role against aphid infestation^37^. Interestingly, in our data the abundance of BES1 family TF genes encoding for starch catabolism such as beta-amylases (*BAM1, BAM3*) were suppressed in LE sample, whereas no differentially expressed genes were observed in case of AC sample. Protein14-3-3 and e2f-dp TF family involved in stress responses were down-regulated in case of LE, similar to rice in which the expression of 14-3-3 protein was high in incompatible than compatible interaction with infecting pathogens^38^.

In Arabidopsis, *Atmyb44* mutants are highly susceptible to *M. persicae* which indicates involvement of MYB44 in defense against aphid^39^. Activation of *MYB44* in response to *A. craccivora* only, supports resistance of *B. juncea* to *A. craccivora*. Similarly, DNA-binding protein AtWRKY22 promotes susceptibility to green peach aphid *M. persicae*, and modulates SA and JA signalling^40^. Global gene expression analysis of *M. persicae*-infested *wrky22* mutants revealed the up-regulation of genes involved in SA signalling and down-regulation of genes involved in plant growth and cell-wall loosening suggesting that WRKY22mediated susceptibility is associated with suppression of SA-signalling^40^. We also observed up-regulation of *WRKY21* in case of *L. erysimi*, and *WRKY19* in *A. craccivora* infestations. Further, the *WRKY70* transcription factors an important node in the convergence between SA and JA signalling was also highly up-regulated in AC sample^41^. One of the TFs associated with WRKY TF family is TGA transcription factors. They are important regulators of SA-induced expression of *PR* genes^42^. TGAs are also implicated in the activation of JA and ET-dependent defense genes in the absence of SA signal^43^. We identified that *TGA4* and *TGA7* were activated in response to *A. craccivora* infestation (Supplementary file 6). Thus, the results suggested involvement of several TFs in nonhost type resistance of *B. juncea* against *A. craccivora*.

ROS such as superoxide, hydrogen peroxide and singlet oxygen are generated during plant responses to both abiotic and biotic stresses^44^. Plants concomitantly generate several ROS scavenging or detoxifying enzymes for preventing cellular damages during oxidative stresses. Expression of several ROS scavenging transcripts was affected in *B. juncea* in response to both the aphid species (Table 3). Down-regulation of larger number of ROS homeostasis related transcripts indicated less propensity of ROS generation in *B. juncea* in case of *L. erysimi* infestation. Similar results were also observed in cotton where infestation by cotton aphid down-regulated several ROS scavenging transcripts and in Arabidopsis, in which aphid-feeding down-regulated several H_2_O_2_ concentration modulating genes^20,45^. In contrary, genes involved in oxidative signal transduction such as peroxidase and catalase were up-regulated in resistant wheat plants infested by the Russian wheat aphid^46^.

The importance of JA pathway in defense against aphids have been demonstrated by induction of JA-dependent pathways by exogenous application of JA^23,47,48^. However, down-regulation of COI1 in LE sample indicated likely disruption in signal transduction process of JA signalling. FAD3 which was activated in LE sample was demonstrated to have an impact on SA- and JA-mediated defense signalling in Arabidopsis^49^. Further, we also observed the involvement of Auxin, ABA and BR pathways during the infestation by *L. erysimi* and *A. craccivora* (Table 4). The role of auxin signalling in plant-aphid interactions is unknown. However, recently it has been shown that PIN5, an auxin transporter involved in regulating intracellular auxin homeostasis and metabolism may be involved in plant susceptibility to aphids as Arabidopsis *pin5* mutants were more resistant to *M. persicae* and *M. cerasi* when compared to wild-type plants^16^. Down-regulation of auxin homeostasis genes *UGT74B1* and *PIN4* by *L. erysimi* whereas all other auxin related transcripts were up-regulated by *A. craccivora*, demonstrated influence of host-defense suppression by *L. erysimi* on the auxin biosynthetic pathway. The role of ABA in plant-aphid interactions has been emerged from studies involving Arabidopsis mutants impaired in ABA biosynthesis or signalling genes. The *M. persicae* fecundity was decreased on the leaves of Arabidopsis mutants that were defective either in ABA synthesis (*aba2*) or in the negative regulation (*abi1*) of the ABA signalling pathways^50^. The role of BR in plant immunity against bacterial pathogens and viruses was demonstrated in mutant Arabidopsis defective in BR signalling by BAK1^51,52^. Further *bak1* mutants of Arabidopsis supported longer survival rate of pea aphid on Arabidopsis which is a nonhost^17^. Mustard aphid down-regulated the expression of BR signalling genes *BAK1* and *BRS1*; however, the BR receptor *BRI* showed increased transcript level (Table 4). In the case of cowpea aphid, for which mustard is a nonhost, all the BR-related genes were down-regulated.

Secondary metabolites play important role in plant defense against pathogens and herbivores^53,54^. The role of flavonoids in plant defense against pathogens, herbivores, and environmental stresses has been well established^55^. Interestingly, all the transcripts coding for flavonoids biosynthesis were down-regulated in case of *L. erysimi* infestation which reinstates the proposition of host defense suppression in case of successful infestation^23^. However, the lignin biosynthesis genes were up-regulated by *A. craccivora*, suggesting the involvement of cell wall refortification to limit its infestation on nonhost *B. juncea*. The biosynthetic genes of phenylalanine-derived lignins and flavonoids were also down-regulated by cabbage aphid, *Brevicoryne brassicae* in Arabidopsis^21^. In chrysanthemum, enhanced expression of lignin biosynthesis genes and lignin accumulation by over-expressing *CmMYB19* transcription factor resulted in limited invasion by the aphids and increased aphid tolerance of chrysanthemum^56^. Along with other secondary metabolites the transcriptome data also indicated more profound suppression of GSL biosynthetic genes by *L. erysimi* (Table 5). Defensive glucosinolates abundant in Brassicaceae members are stored in specialized cells and when tissue damage occurs, they are hydrolysed by myrosinases to produce various products which are toxic and or deterrents to herbivores^57^. In LE sample, we observed down-regulation of the transcripts related to biosynthesis of aliphatic glucosinolates. Similar results were observed in Arabidopsis where infestation by aphids down-regulated GSL-metabolic genes^21,58^. The transcripts of myrosinase (*TGG1*), involved in breakdown of glucosinolates and *NSP2*, involved in glucosinolate hydrolysis with the help of myrosinase^59^ were also differentially regulated by both the aphids in *B. juncea*. It was intriguing to note that the cowpea aphid *A. craccivora* had a little impact on the expression of glucosinolate biosynthesis and breakdown genes, suggesting a possible role of glucosinolate-myrosinase pathway in limiting *A. craccivora* from colonizing on *B. juncea* plants.

## Conclusion

Members of Brassicaceae family including rapeseed-mustard are rich reservoir of defensive phytochemicals including glucosinolates^60^. While these defensive metabolites are responsible for resistance to a large number of herbivores and pathogens, mustard aphids, a specialist aphid species rapidly colonize most of the rapeseed-mustard crops. Thus, it was intriguing to identify the mechanistic differences in defense activation when the *B. juncea* plants deter an aphid species as not being a natural host of it. Identification of important genes and pathways leading to nonhost defense vis a vis their counter suppression in case of susceptibility as host is likely to provide important clues for developing varietal resistance. In future perspective, the present work supplemented the limited resource of transcriptome base, much needed to validate various defense pathways and their differential regulations under host-type and nonhost-type defense response, in *B. juncea*.

## Materials and Methods

### Plant material and insect-infestations

Growing conditions of Indian mustard, *Brassica juncea* cv. Varuna and maintenance of mustard aphids were carried out as described previously by Koramutla *et al*.^23^. The cowpea aphid, *Aphis craccivora* was maintained on cowpea plants grown and maintained in a growth chamber, set at 24 ± 1 °C, 65–70% relative humidity and 16/8 h (light/dark) photoperiod. Four-week old *B. juncea* plants were used for aphid-infestation experiments. One hundred adult aphids on each plant were released for infestation and allowed to settle and feed on the plants. After 24 h of infestation, aphids were removed gently with the help of a paint brush, leaf samples were collected in liquid N_2_ and stored at -80 °C until further analysis. Similarly, mock brushed leaves were collected from the uninfested plants as controls.

### Aphid performance on *B. juncea*

To evaluate the performance of the aphid species *L. erysimi* and *A. craccivora* on the mustard plants, five adult aphids were confined on a leaf using clip-cage. Total number of nymphs produced, and survival of the adult aphids were recorded after 4 days post infestation. The experiments were performed independently on three biological and five technical replicates. The data was analyzed on Microsoft excel using one-way ANOVA, mean separations and significance were tested using Student’s *t*-test (*p* < 0.05).

### Ninhydrin staining and quantification of aphid honeydew

*B. juncea* leaves were infested with 100 individuals each of *L. erysimi* (LE) and *A. craccivora* (AC) in independent experiments. Honeydews were collected on the 3MM whatman paper discs in Petri dishes (90 × 15 mm), placed under infested *B. juncea* leaves. Similar arrangement was replicated for uninfested *B. juncea* leaves which served as controls. After 24 h of infestation, whatman papers were collected and soaked in 0.1% (w/v) ninhydrin prepared in acetone and dried at 65 °C in a hot air oven for 30 min^24^. The whatman paper discs with purple colour spots were scanned for documentation. For quantifying the aphid honeydews, the paper discs were cut into pieces and the stains were extracted in 10 ml of 90% (v/v) methanol for 1 h with periodical shaking. The samples were centrifuged at 6000 rpm for 5 min. and the supernatants were measured at 500 nm against 90% methanol as blank^24^.

### Library construction and deep sequencing

Total RNA was extracted from leaf samples infested with aphids for 24 h using RaFlex total RNA Isolation kit (GeNei, India) according to the manufacturer’s instructions. The total RNA pooled from three biological replicates were used for library preparation, sequencing and unigene identification outsourced to Xcelris Labs Limited (www.xcelrisgenomics.com). The paired-end cDNA sequencing library was prepared using Illumina TruSeq RNA Library Preparation V2 Kit as per manufacturer’s protocols. Briefly, mRNA was enriched and fragmented enzymatically. These short fragments were used for first and second strand cDNA synthesis, followed by end repair, A-tailing and adapter ligation, and finally to index PCR amplification of adaptor-ligated library. Library quantification and qualification was performed by using a HT DNA High Sensitivity Assay kit. The mean fragment sizes of the libraries were in the range of 450–675 bp. Finally, the library was sequenced using Illumina MiSeq/NextSeq.

### Bioinformatics analyses

The raw reads obtained from the Illumina were filtered to exclude low quality reads and the reads containing adaptor sequences. The resulting clean reads were assembled separately for each library with CLC Genomics Workbench (version 6.0). The assembled contigs were validated by mapping reads back to the assembled contigs. The coding sequences (CDS) were predicted from Control, LE and AC-infested assembled contigs using ORF-Predictor with default parameters. The predicted CDS were annotated by blastx^61^ against the NCBI non-redundant or The Arabidopsis Information Resource (TAIR10) protein databases with an E-value threshold of < 1e-6. Blast2GO program^62^ was used for Gene Ontology (GO) and KEGG annotation of the CDS^25^. The calculation of transcript expression used the FPKM method. After FPKM calculation, common hit accessions based on BLAST against non-redundant databases were identified for differential gene expression analysis. The transcripts whose log2 ratio ±2 (four-fold change) and *P* < 0.05 between the uninfested control and aphid-infested samples were considered as differentially expressed. The differentially expressed genes (DEGs) were subjected to analysis of metabolic pathways and plant transcription factors. The MapMan application software was used to visualize the DEG involved in the metabolic pathways^63^.

### Validation of gene expression using qRT-PCR

Twelve differentially expressed genes were selected for validation using qRT-PCR. The primers were designed using IDT primer quest software (https://www.idtdna.com/). cDNA was synthesized from DNase treated total RNA (2 µg) using PrimeScript 1^st^ strand cDNA synthesis kit (TaKaRa Bio Inc, Japan) as per the manufacturer’s instructions and diluted 20 times with nuclease free water. The qRT-PCR was performed on StepOne Plus Real-Time PCR (Applied Biosystems, USA) in a final volume of 20 µL containing 2 µL diluted cDNA, 10 µL 2xSYBR Premix Ex Taq (TaKaRa Bio Inc, Japan), 0.4 µL ROX reference dye, 0.4 µL each of forward and reverse primer (10 µM), and 6.8 µL RNase-free water as described in Koramutla *et al*.^64^. The thermal cycling conditions were as follows: 95 °C for 1 min followed by 40 repeated cycles of 95 °C for 10 s, 60 °C for 30 s, and 72 °C for 30 s. Relative gene expression was determined using 2^−ΔΔCT^ method by normalizing to the *GAPDH* gene expression. The primers used for qRT-PCR validation were listed in Supplementary Table S1.

## Supplementary information

Supplementary Information.

Supplementary Information 2.

Supplementary Information 3.

Supplementary Information 4.

Supplementary Information 5.

Supplementary Information 6.

Supplementary Information 7.

Supplementary Information 8.

Supplementary Information 9.

Supplementary Information 10.

Supplementary Information 11.
