# Supplementary material for: Comparative transcriptomics revealed differential regulation of defense related genes in Brassica juncea leading to successful and unsuccessful infestation by aphid species
Source: Sci Rep. 2020 Jun 29;10:10583. doi: 10.1038/s41598-020-66217-0 (PMC7324606; doi:10.1038/s41598-020-66217-0)

Fig S2A . Enriched biological process related GO terms in the *A. craccivora* infested *B. juncea.* The GO terms are represented with in the boxes and the different colors indicates the significance level of GO terms.

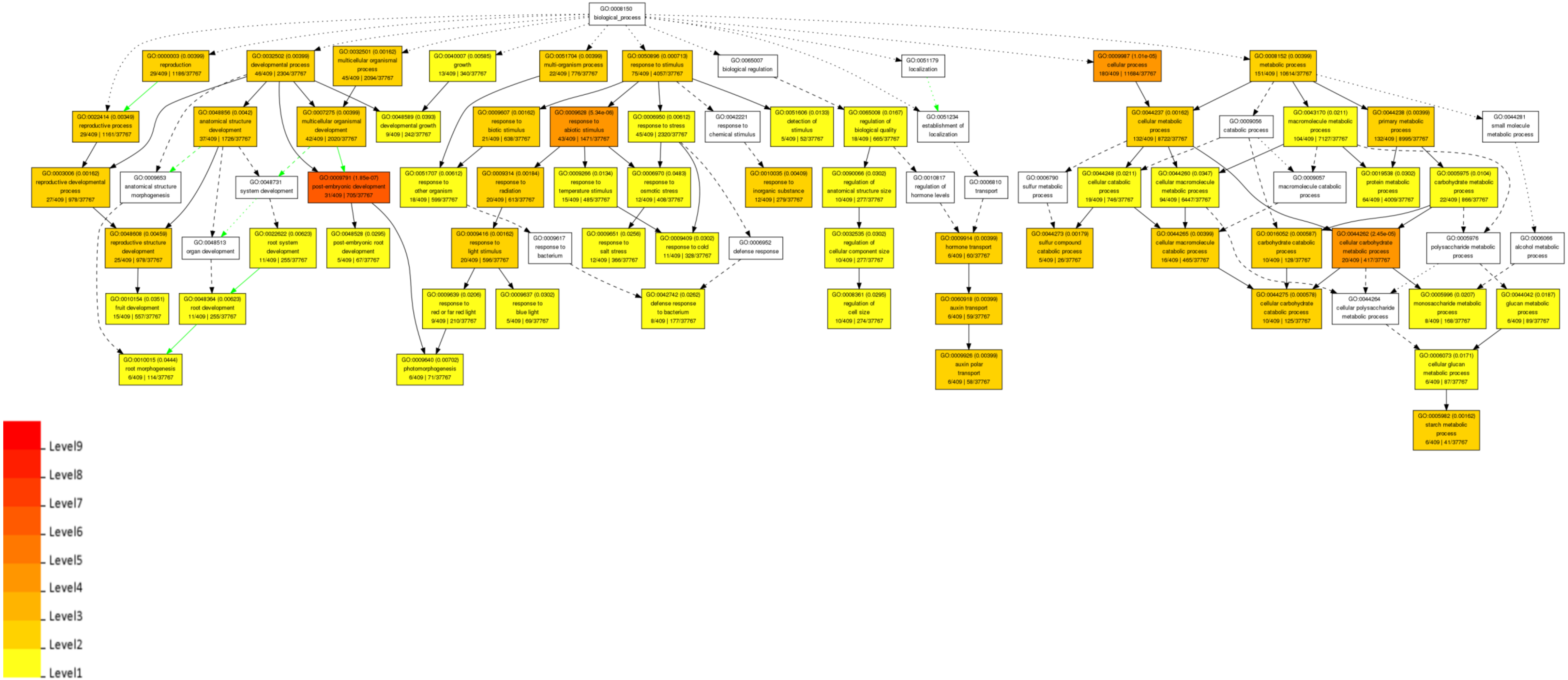

Fig S2B . Enriched molecular process related GO terms in the *A. craccivora* infested *B. juncea.* The GO terms are represented with in the boxes and the different colors indicates the significance level of GO terms.

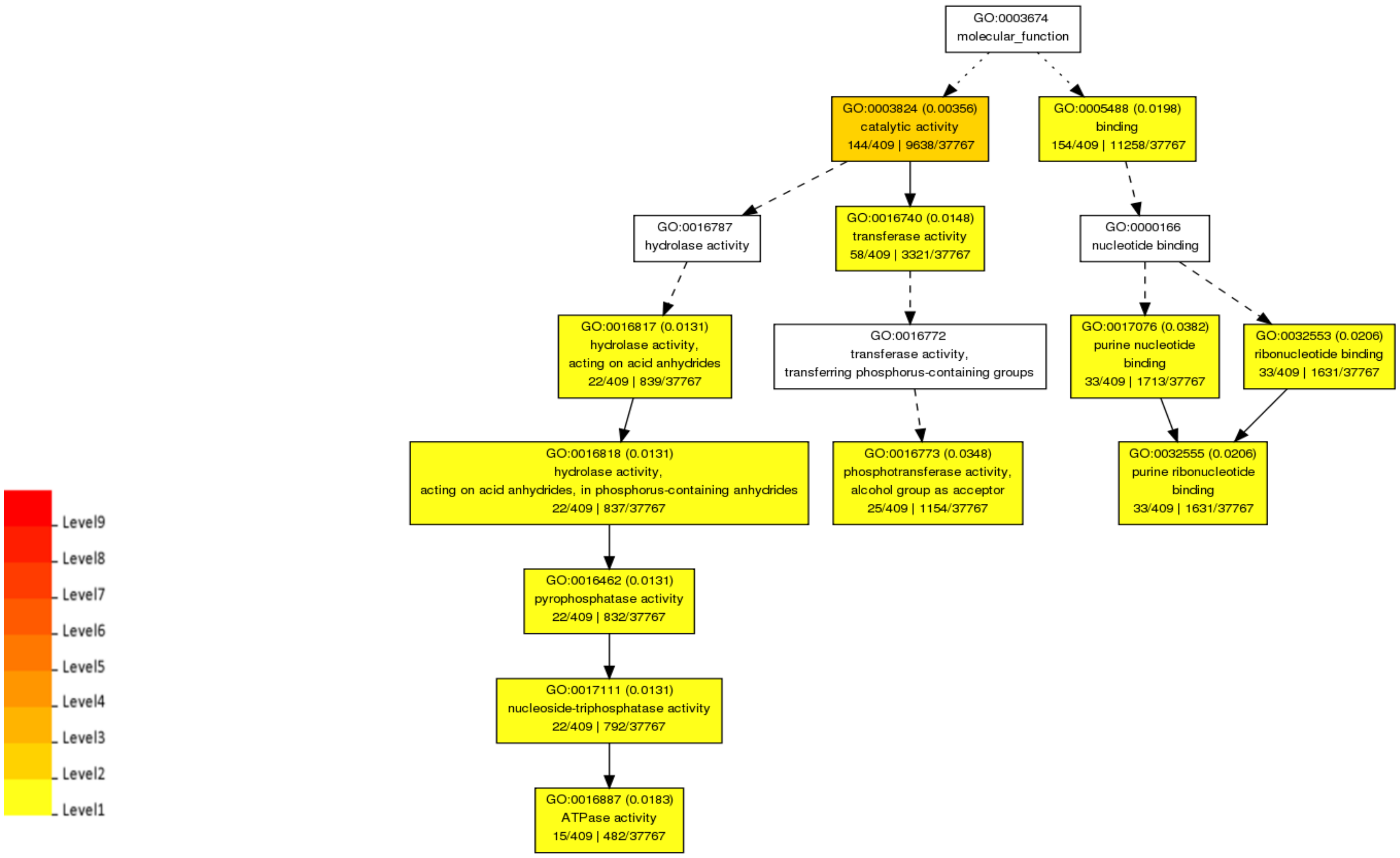

Fig S2C. Enriched cellular process related GO terms in the *A. craccivora* infested *B. juncea*. The GO terms are represented with in the boxes and the different colors indicates the significance level of GO terms.

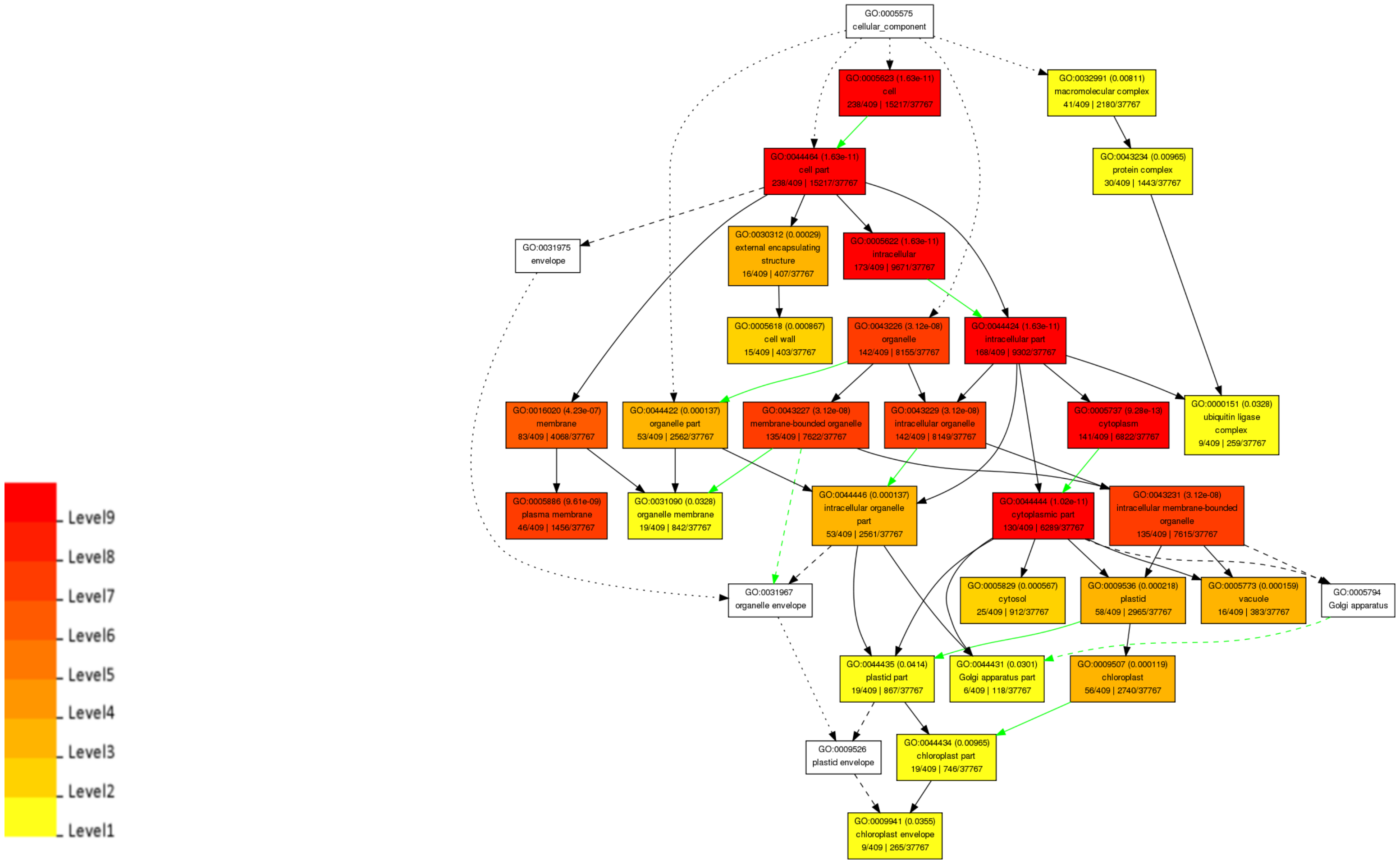

Fig S2D. Enriched biological process related GO terms in the *L. erysimi* infested *B. juncea*. The GO terms are represented with in the boxes and the different colors indicates the significance level of GO terms.

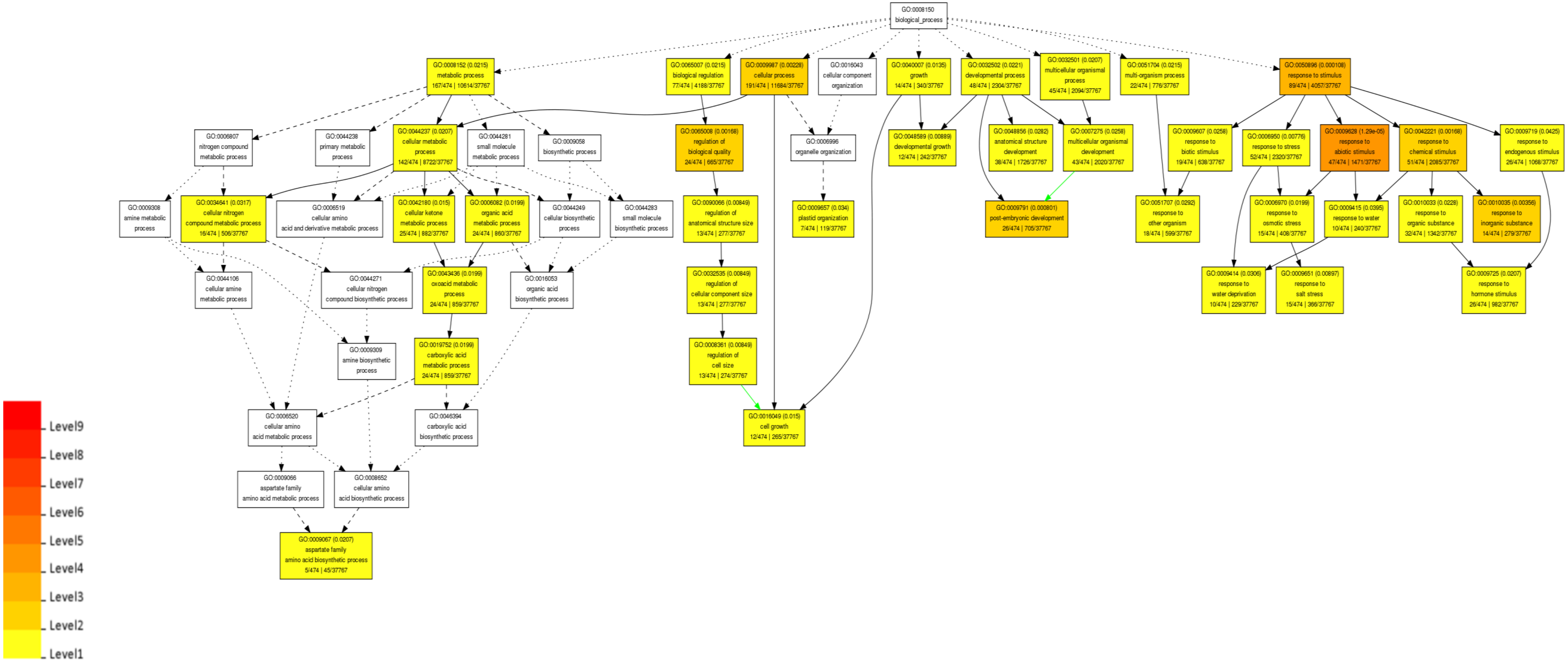

Fig S2E. Enriched molecular process related GO terms in the *L. erysimi* infested *B. juncea*. The GO terms are represented with in the boxes and the different colors indicates the significance level of GO terms.

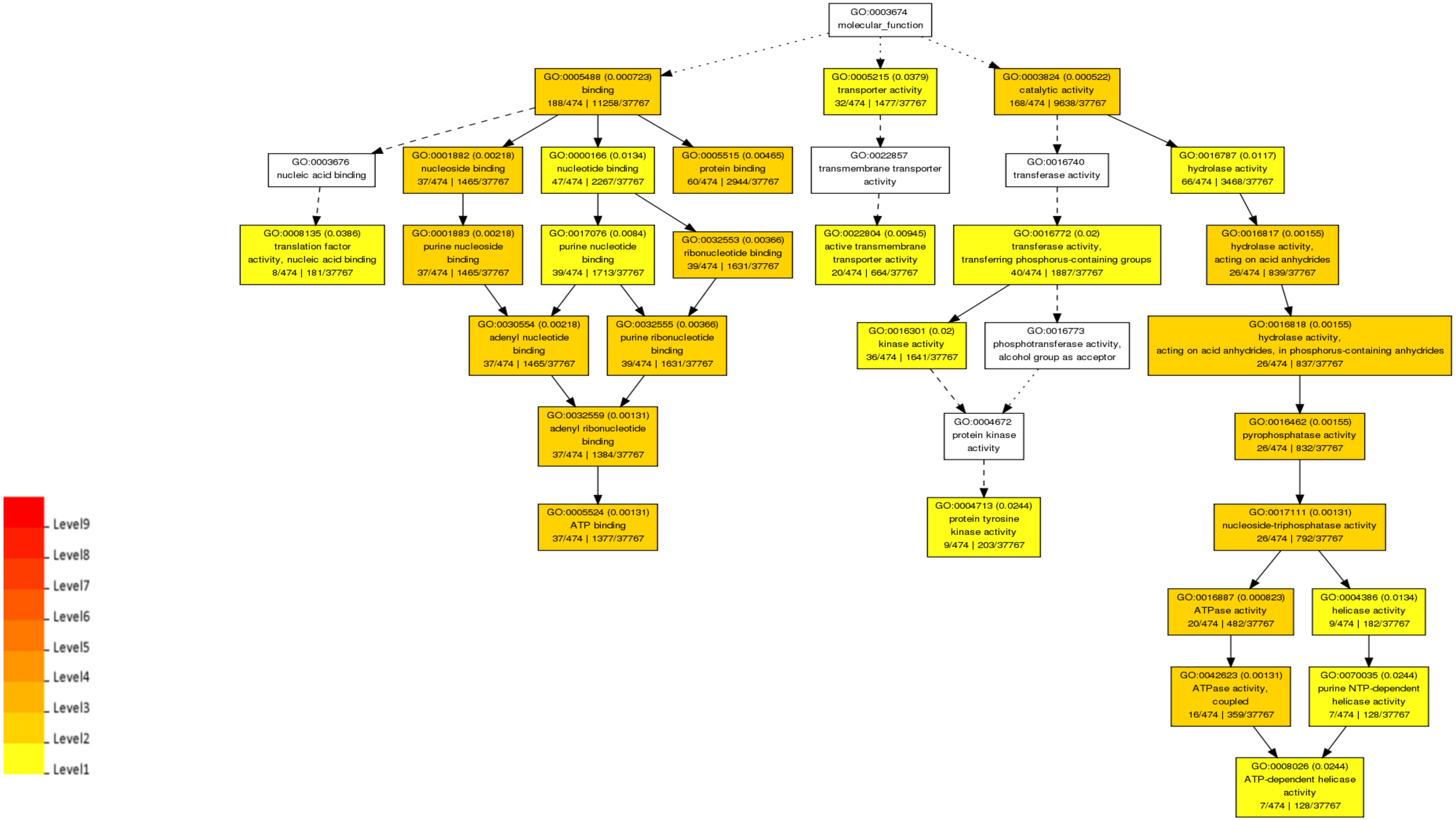

Fig S2F. Enriched cellular process related GO terms in the *L. erysimi* infested *B. juncea*. The GO terms are represented with in the boxes and the different colors indicates the significance level of GO terms.

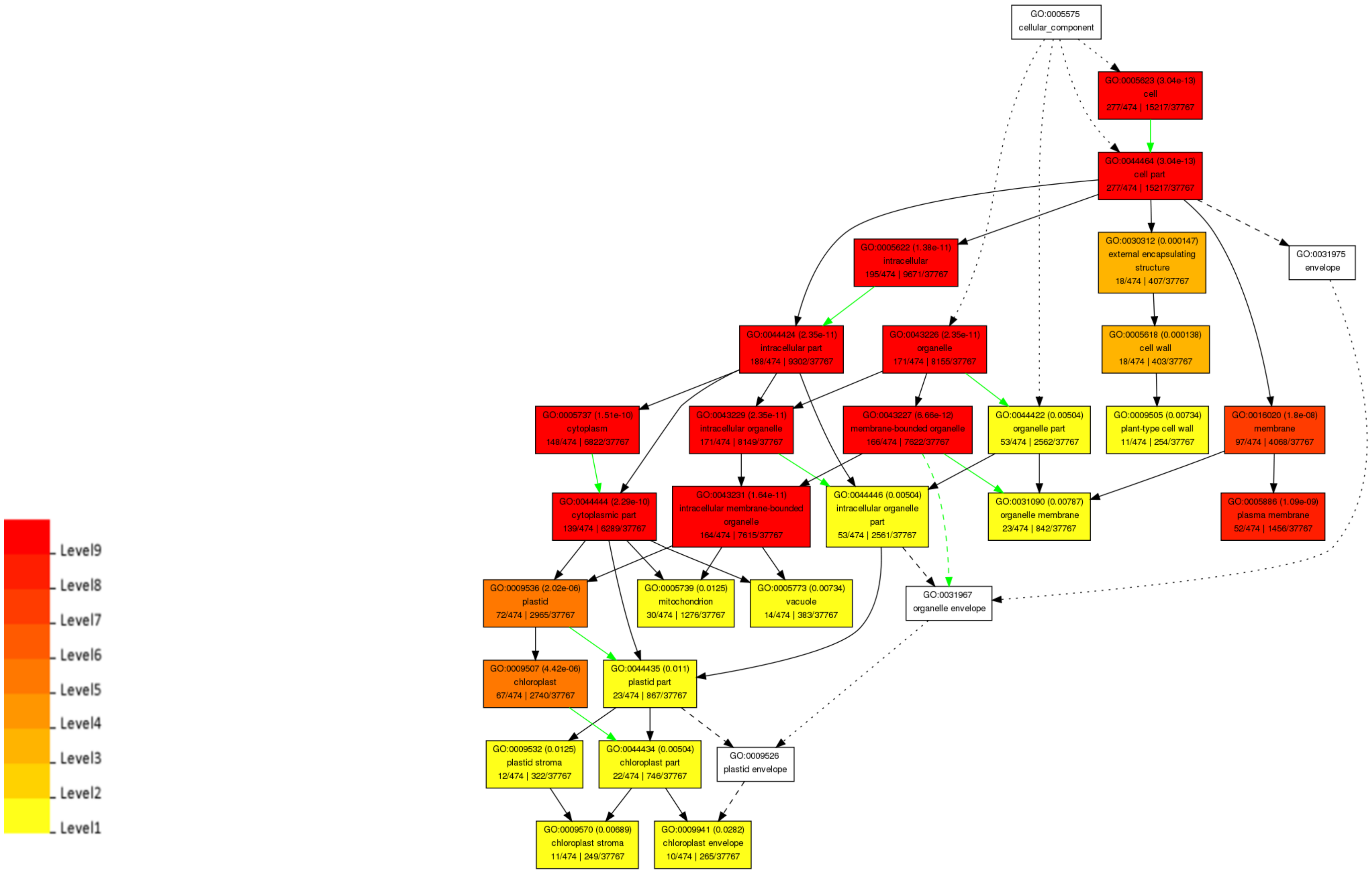

Supplement: Supplementary file 2 — Supplementary Information 2. [file 41598_2020_66217_MOESM2_ESM.pdf]
