# Supplementary material for: Comparative transcriptomics revealed differential regulation of defense related genes in Brassica juncea leading to successful and unsuccessful infestation by aphid species
Source: Sci Rep. 2020 Jun 29;10:10583. doi: 10.1038/s41598-020-66217-0 (PMC7324606; doi:10.1038/s41598-020-66217-0)
Supplement: Supplementary file 9 — Supplementary Information 9. [file 41598_2020_66217_MOESM9_ESM.docx]

**Supplementary Table S1: List of primer pairs used for qRT-PCR validation**

| **Common hit ID** | **Forward primer (5'-3')** | **Reverse primer (5'-3')** | **Amplicon**  **Size (bp)** | **TAIR ID** | **TAIR Description** |
| --- | --- | --- | --- | --- | --- |
| ESQ54135 | CTCTTGCGTAGACGGTTTGT | AGTGACGTTAGCGTGCATAG | 108 | at4g30610 | BRI1 suppressor 1 (BRS1) |
| NP_189093 | CCGGTGATCAACACGAAGAA | CTCCAACCGAGCTTTCTGTATC | 102 | at3g24500 | Multiprotein bridging factor 1C (MBF1C) |
| ESQ30852 | GGCTGAGCTCAAACCCTAAA | TACGTGCTCCCTTTCTTGTTC | 101 | at5g23960 | Terpene synthase 21 (TPS21) |
| BAJ34562 | ACGTGGACGATGTACCAATAAG | GGCTTCCCATAGTGTTGAGTAA | 104 | at5g65730 | Xyloglucan endotransglycosylase, putative |
| XP_002888923 | CGAGATGCAAACACACAAGAC | TGGTGTTGAGTGGAGAGAAAG | 103 | at1g73325 | Trypsin and protease inhibitor family protein |
| AAD17935 | GTCCGTTTCTCCACCGTTAT | CAAAGTTCCCTTCTCTGGTGTA | 100 | at4g35090 | Catalase 2 (CAT2) |
| AGD95055 | ACTGAAGACGACGTGAAGAAC | ATGCAAGCACGAGGGATAAG | 101 | at4g13770 | Cytochrome P450 83A1 (CYP83A1) |
| BAJ33811 | GACCAAGGAACGGACCTATTG | CAACGTCGAACTTGGGTGTA | 142 | at3g03190 | Glutathione S-transferase F11 (GSTF11) |
| BAJ34136 | GATCGCTGCAAATTGGATCAC | GGTGGAAGAAACCGAAGAAGA | 142 | at5g04150 | Transcription factor bHLH101 (bHLH101) |
| NP_568152 | CTAGTGCACCGTGGCATATT | TCTTCCTCCTCTCTGTTTCTCT | 159 | at5g05250 | Hypothetical protein |
| BAJ34294 | GGGAATCAGACTCTCACCATTT | ACGCCATGCTTGTTCATTTC | 102 | at1g76690 | 12-oxophytodienoate reductase 2 (OPR2) |
| ESQ27019 | GATTCCTGAGTGGTGGAGATG | GGCTTGTGCTGTTATCGAATG | 128 | at1g59870 | ABC transporter G family member 36 (ABCG36) |
| - | TCAGTTGTTGACCT CACGGTT | CTGTCACCAACGAAGTCAGT | 100 | at3g04120 | Glyceraldehyde-3-phospho dehydrogense (GAPDH) |
