# Supplementary material for: Comparative transcriptomics revealed differential regulation of defense related genes in Brassica juncea leading to successful and unsuccessful infestation by aphid species
Source: Sci Rep. 2020 Jun 29;10:10583. doi: 10.1038/s41598-020-66217-0 (PMC7324606; doi:10.1038/s41598-020-66217-0)
Supplement: Supplementary file 10 — Supplementary Information 10. [file 41598_2020_66217_MOESM10_ESM.doc]

**Supporting Information**

**Comparative transcriptomics revealed differential regulation of defense related genes in *Brassica juncea* leading to successful and unsuccessful infestation by aphid species**

Lianthanzauva Duhlian^1^, Murali Krishna Koramutla^1^, S. Subramanian^2^, Rohit Chamola^1^, Ramcharan Bhattacharya^1^*

^1^ICAR-National Institute for Plant Biotechnology, Indian Agricultural Research Institute Campus, New Delhi 110012, India.

^2^Division of Entomology, Indian Agricultural Research Institute, New Delhi 110012, India.

* Corresponding author.

E-mail address: rcbhattacharya1@gmail.com (R. BHATTACHARYA).
