# Supplementary material for: Comparative transcriptomics revealed differential regulation of defense related genes in Brassica juncea leading to successful and unsuccessful infestation by aphid species
Source: Sci Rep. 2020 Jun 29;10:10583. doi: 10.1038/s41598-020-66217-0 (PMC7324606; doi:10.1038/s41598-020-66217-0)
Supplement: Supplementary file 11 — Supplementary Information 11. [file 41598_2020_66217_MOESM11_ESM.docx]

**Supporting information**

Following supplementary data may be available online in the supporting information tab.

Supplementary Table S1. List of primer pairs used for qRT-PCR validation

Supplementary file 1. GO classification of uninfested *B. juncea* leaf transcriptome

Supplementary file 2. GO classification of *L. erysimi* infested *B. juncea* leaf transcriptome

Supplementary file 3. GO classification of *A. craccivora* infested *B. juncea* leaf transcriptome

Supplementary file 4. Differentially expressed transcripts in response to infestation by *L. erysimi* and *A. craccivora* in comparison to the uninfested control

Supplementary file 5. GO terms of the differentially expressed transcripts in *L. erysimi* and *A. craccivora* infested samples over the control

Supplementary file 6. Differentially expressed transcription factors in *B. juncea* in response to *L. erysimi* and *A. craccivora*

Supplementary Fig. S1. Transcript and CDS length in leaf transcriptomes of *Brassica juncea*.

Supplementary Fig. S2. Enriched GO terms of differentially expressed transcripts in *B. juncea* in response to *L. erysimi* and *A. craccivora*
